# The identification of trans-associations between prostate cancer GWAS SNPs and RNA expression differences in tumor-adjacent stroma

**DOI:** 10.18632/oncotarget.2763

**Published:** 2015-02-09

**Authors:** Xin Chen, Michael McClelland, Zhenyu Jia, Farah B. Rahmatpanah, Anne Sawyers, Jeffrey Trent, David Duggan, Dan Mercola

**Affiliations:** ^1^ Genomics Center, Loma Linda University, Loma Linda, California, 92354, United States of America; ^2^ Department of Pathology and Laboratory Medicine, University of California, Irvine, California, 92697, United States of America; ^3^ Department of Microbiology and Molecular Genetics, University of California, Irvine, California, 92697, United States of America; ^4^ Department of Statistics, The University of Akron, Akron, Ohio, 44325, United States of America; ^5^ Department of Family & Community Medicine, Northeast Ohio Medical University, Rootstown, Ohio, 44272, United States of America; ^6^ Genetic Basis of Human Disease Division, The Translational Genomics Research Institute, Phoenix, Arizona, 85004, United States of America; ^7^ Integrated Cancer Genomics Division, The Translational Genomics Research Institute, Phoenix, Arizona, 85004, United States of America

**Keywords:** SNPs, eQTL, prostate cancer

## Abstract

Here we tested the hypothesis that SNPs associated with prostate cancer risk, might differentially affect RNA expression in prostate cancer stroma. The most significant 35 SNP loci were selected from Genome Wide Association (GWA) studies of ~40,000 patients. We also selected 4030 transcripts previously associated with prostate cancer diagnosis and prognosis. eQTL analysis was carried out by a modified BAYES method to analyze the associations between the risk variants and expressed transcripts jointly in a single model. We observed 47 significant associations between eight risk variants and the expression patterns of 46 genes. This is the first study to identify associations between multiple SNPs and multiple in trans gene expression differences in cancer stroma. Potentially, a combination of SNPs and associated expression differences in prostate stroma may increase the power of risk assessment for individuals, and for cancer progression.

## INTRODUCTION

Prostate cancer is the most frequently diagnosed male cancer and the second leading cause of cancer death in men in the United States [1]. However, only a fraction of cases of prostate cancer lead to death. Thus, reliably identifying individuals at higher risk of progression to metastatic disease is of great potential utility.

GWA (Genome-wide association) studies have been performed to identify more than 70 risk variants associated with overall risk of developing prostate cancer [2–13]. A few recent GWA studies showed that some of these risk variants may correlate with the progression of prostate cancer [14–19] and, thus, could be clinically useful given that the majority prostate cancer cases are indolent and not a threat to life. Individually, the identified risk variants are low-penetrance susceptibility loci and have little clinical utility. Almost all are located in non-protein-coding or intergenic regions with unknown mechanisms of influence on phenotype. GWA follow-up studies have identified possible associations between the risk variants and gene expression levels of nearby or local genes (cis-associations) [6, 10, 13]. Remote associations (trans-) between these SNP variants and significant expression changes are likely but unknown, as yet.

Prostate cancer risk alleles can ultimately manifest their phenotype in a variety of ways. A subset may do so by altering gene expression in one or more cell types, including prostate epithelium, prostate stroma, the immune system, or others. In addition, an overlapping subset of risk SNPs may also affect progression after cancer has arisen, mediated by expression in one or more cell types.

Independent of SNP data, progression risk within the tumors has been associated with differences in gene expression [20–23] and in DNA methylation [24–26]. However, the cell, genetic, and epigenetic heterogeneity within a tumor and between tumors is a barrier to developing biomarkers for reliable prognosis. The number of candidate biomarkers that agree across studies is very small [23]. Similarly, mapping the effect of SNPs on expression in tumor could also be noisy. Reactive stroma initiates during early prostate cancer development and coevolves with prostate cancer progression. Recent evidence suggests that prostate epithelium and stroma interact in a highly organ-specific, androgen-dependent, and temporally related manner [27, 28]. Meanwhile, few somatic genetic differences have been identified in the stroma of prostate cancer [29, 30] and we have shown that the stroma undergoes expression differences with diagnostic [31] and prognostic potential [32].

If SNP risk markers could be linked with diagnostic and prognostic expression data in the relatively genetically stable environment of the stroma then, together, these associations may increase the utility of low risk SNPs by identifying a subset of patients where an otherwise low penetrance risk SNP was relevant.

To make the first step towards this goal, we applied eQTL (Expression Quantitative Trait Loci) analysis to expression data from tumor-adjacent stroma, to define associations between gene activity and risk variants [33–36]. This is a powerful method that treats each gene as a quantitative trait and identifies loci whose genotype variation such as in SNPs is associated with gene expression difference. The identified loci are considered as eQTLs.

In the eQTL analysis, we modified a Bayesian clustering method [34] to analyze expressed prostate cancer-related genes and susceptible loci jointly in a single model. Applying the approach to stroma-enriched samples, we identified 47 eQTL associations. In particular, the variant rs10896449 is associated with 32 significant expression differences in the stroma. This is the first study to identify trans-eQTL associations occurring between multiple SNPs and multiple significant expression differences in cancer stroma. The possible relationships between the identified associations and clinical properties, including cancer outcome, is examined and trends are identified.

## RESULTS

### Identification of SNP-transcript associations

A literature search of prostate cancer-related genes was conducted through the electronic database PubMed [20–23, 31, 32, 37–39] including differential expressed genes of utility for diagnosis and prognosis and genes with local distance to susceptible loci as reported in GWA studies. A total of 4030 such genes were identified. A second literature search of GWA studies of prostate cancer was conducted through the electronic database PubMed [2–14, 18, 40–43] covering a total of ~40000 prostate cancer cases, identifying more than 70 susceptible SNPs that were reported to have significant association with prostate cancer. 35 SNPs were selected and summarized in Supplementary Tables S2 and S3 for eQTL analysis.

In eQTL analysis, we examined possible associations between the 35 SNPs and 4030 transcripts using 49 stroma-enriched samples. Four eQTL analyses were performed in total. Samples with stroma cell type percentage greater than 50%, 60%, 70%, and 80% were used for the four eQTL analyses. Table 1 shows the number of selected samples in the four eQTL analyses. In addition to the “80%” criterion described above, an eQTL was only accepted when the transcript-SNP association appeared in *all four* eQTL analyses.

**Table 1:** Number of samples used in four eQTL analyses on the basis on varying stroma cell type percentage

Stroma percentage	>50%	>60%	>70%	>80%
Number of samples	49	41	33	25

A total of 47 associations including 8 SNPs and 46 transcripts were identified in all four eQTL analyses. Table 2 shows in detail the combinations of associations among 8 SNPs and 46 transcripts. For example, the SNP rs10896449 is associated with the most transcripts, 32 transcripts.

**Table 2:** 47 associations between 8 SNPs and 46 transcripts in eQTL analysis

Association ID	Probe Set	Gene Symbol	Location
**SNP rs10896449 (G) 11q13**			
1	209716_at	*CSF1*	1p13
2	204175_at	*ZNF593*	1p36
3	204197_s_at	*RUNX3*	1p36
4	202546_at	*VAMP8*	2p11-p12
5	205174_s_at	*QPCT*	2p22
6	218864_at	*TNS1*	2q35-q36
7	226766_at	*ROBO2*	3p12
8	219551_at	*EAF2*	3q13
9	232099_at	*PCDHB16*	5q31
10	205100_at	*GFPT2*	5q34-q35
11	208583_x_at	*HIST1H2AJ*	6p22
12	223475_at	*CRISPLD1*	8q21
13	204501_at	*NOV*	8q24
14	205041_s_at	*ORM1 /// ORM2*	9q32
15	205127_at	*PTGS1*	9q32-q33
16	213004_at	*ANGPTL2*	9q34
17	203666_at	*CXCL12*	10q11
18	204396_s_at	*GRK5*	10q26
19	203835_at	*LRRC32*	11q13-q14
20	211964_at	*COL4A2*	13q34
21	201562_s_at	*SORD*	15q15
22	203151_at	*MAP1A*	15q15
23	214297_at	*CSPG4*	15q24
24	224476_s_at	*MESP1*	15q26
25	229730_at	*SMTNL2*	17p13
26	218980_at	*FHOD3*	18q12
27	37996_s_at	*DMPK*	19q13
28	222106_at	*PRND*	20p13
29	205439_at	*GSTT2*	22q11
30	201787_at	*FBLN1*	22q13
31	220663_at	*IL1RAPL1*	Xp21-p22
32	204584_at	*L1CAM*	Xq28
**SNP rs1859962 (G) 17q24**			
33	238079_at	*TPM3*	1q21
34	206307_s_at	*FOXD1*	5q12-q13
35	203438_at	*STC2*	5q35
36	205040_at	*ORM1*	9q32
**SNP rs401681 (C) 5p15**			
37	206529_x_at	*SLC26A4*	7q31
38	204846_at	*CP*	3q23-q25
39	203021_at	*SLPI*	20q12
**SNP 9623117 (C) 22q13**			
40	206307_s_at	*FOXD1*	5q12-q13
41	220120_s_at	*EPB41L4A*	5q21
42	228256_s_at	*EPB41L4A*	5q21
43	214676_x_at	*MUC3A*	7q22
**SNP rs12621278 (G) 2q31**			
44	211734_s_at	*FCER1A*	1q23
**SNP rs1465618 (A) 2p21**			
45	205132_at	*ACTC1*	15q14
**SNP rs620861 (C) 8q24**			
46	210452_x_at	*CYP4F2*	19p13
**SNP rs6983267 (G) 8q24**			
47	223775_at	*HHIP*	4q28-q32

In order to test whether any of the 47 associations are false-positives, we carried out two extensive resampling studies in order to estimate false discovery rates for the associations. The first resampling study is a permutation test as follows:

1. Permute SNP samples for the smallest data set (>80% stroma).
2. Move to the next smallest data set and permute the SNP samples which are not included in previous permutated data set.
3. Repeat step 2 until all four data sets are permuted.

After permutation, the modified BAYES method was used to perform eQTL analyses between the randomly permutated SNP data and original expression data. The associations that appear in all four eQTL analyses were considered as false discovered associations. A total of 1000 permutations were performed. No permutation was identified with associations greater than 47 associations. The average number of falsely discovered associations is 1.57 compared to the 47 observed, and a false discovery rate of 3.3% was estimated.

In the second resampling study, 35 SNPs were randomly selected from over 1 million SNPs represented on the Illumina SNP array and used to perform four eQTL analyses. Again a total of 1000 random selections were performed. Again no permutation was identified with associations greater than 47 associations. The average number of falsely discovered associations is 2.55 compared to 47 observed, and the false discovery rate was estimated as 5.4%. Thus, the two resampling studies are consistent and strongly indicate that only three of our identified 47 transcript-SNP associations could be considered to occur by chance. The genes of the significant expression differences linked to 8 SNPs are given in Table 2.

### Association of SNP-transcript associations with clinical parameters

We followed a two-step procedure (see Supplementary methods for details) to boost the power of identifying possible associations with clinical properties. This was necessary because the power to identify highly significant correlations with clinical parameters is limited due to the small sample size which needs to be further divided into subgroups for such an analysis.

**Figure 1:**
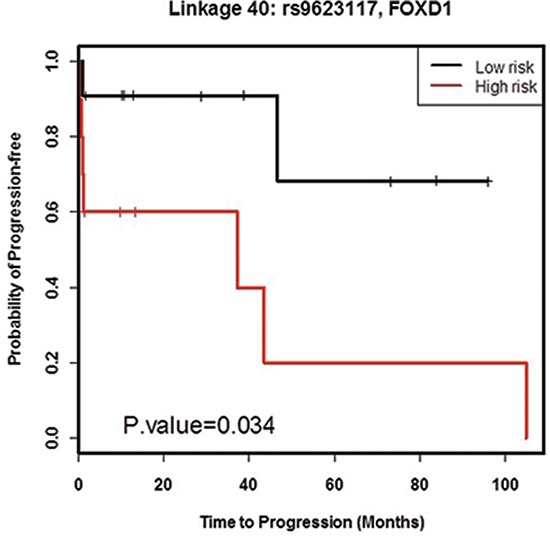
Survival analysis of high and low risk groups defined by FOXD1-rs9623117

First, the 49 patients were subdivided into three *risk groups* (high, intermediate, and low risk group) defined by each transcript-SNP association. Supplementary Figure 1 shows the way that the three risk groups were defined. Then ordinal logistic regression analysis was performed to identify associations between risk groups and clinical properties. Supplementary Table S3 shows the results of ordinal logistic regression analysis. Association 40 (*FOXD1* and rs9623117 C) is the most interesting association because the *p*-values in both univariate and multivariate models were less than 0.05. Furthermore, we examined the high and low risk groups only defined by association 40. A Kaplan-Meier survival analysis (Figure 1) has an estimated hazard ratio 2.23 and significant *p*-value of 0.034 (logrank test).

## DISCUSSION

In previous studies, gene signatures were identified by gene expression profiling and genetic variants were identified by GWA studies. These results were used to develop biomarkers for prostate cancer diagnosis and prognosis. However, the low concordance among gene signatures of profiling studies and the marginal risk of the best loci found in GWA studies have reduced the utility of such markers, to date. We speculated that by combining expression data with known genotypes, the resulting associations would add value and provide new evidence of a mechanism for the association between SNP and risk of prostate cancer.

We have established significant associations between risk-associated SNPs and RNA expression levels in tumor-adjacent prostate stroma. We used tumor-adjacent stroma instead of tumor for the study because there are very few genetic alternations in stroma [29, 30, 44, 45]. Studying the stroma does not preclude observing eQTLs in the tumor however. On the contrary, variants affecting expression in the tumor can in turn affect expression in the stroma because there are many active interactions between tumor and tumor-adjacent stroma in prostate cancer [31, 46, 47]. Epithelial cells of prostate cancer infiltrate and propagate in a microenvironment consisting largely of myofibroblast cells as well as inflammatory cells and other supporting cells and structures. The mesenchymal component is not passive but responds to signals from the tumor component and, in turn, alters tumor properties, some of which are essential for tumor growth and progression [46, 48]. Similarly SNPs affecting the endocrine and immune system may also be detectable in the response of prostate stroma.

We modified the BAYES method to perform an eQTL study to identify potential associations between risk loci and genes differentially expressed in prostate cancer stroma. The comprehensive mapping between SNPs and transcripts is one of the advantages of the modified BAYES method, allowing the eQTL study to identify not only cis-linkages but also trans-linkages. We utilized an extensive amount of information that is available for prostate cancer from prior GWA studies, encompassing around 40,000 patients, in order to form a short list of 35 SNPs for further consideration. Similarly a short list of 4030 prostate cancer-related genes were selected based on previously reported prostate cancer diagnosis and prognosis studies. To reduce false discovery rate, we performed eQTL analysis on four data sets with increased stroma percentages. Our two permutation tests also showed that the false discovery rate is very low. We obtained both expression and SNP data for 49 patients, 27 non-relapse, 20 relapse, and 2 unknown (Supplemental Table S1 for list of parameters) and observed 47 associations between eight SNPs and 46 transcripts (45 genes).

Next we examined if there was any evidence that the SNP-expression associations could better stratify patients for risk of progression of prostate cancer. Most SNPs associated with prostate cancer have been identified in case-control GWA studies. However, SNPs associated with disease progression are beginning to be identified. A recent study reported that two variants at 10q11 and one variant at 8q24 associated with susceptibility influence biochemical recurrence following radical prostatectomy [49]. Variants at 8q24 have been evaluated for risks of aggressiveness of prostate cancer based on age at onset, familial aggregation and tumor grades and stages. Some GWA studies have reported that the aggressive forms of the disease are influenced by the same variants associated with susceptibility to prostate cancer [15, 16, 18, 19]. A recent study also showed that variants at 8q24 act as enhancers of proto-oncogene c-MYC [50]. c-MYC is also located at 8q24, a known regulator of cell growth, and has a critical role in prostate cancer development and progression. It is likely that a subset of SNPs affecting the probability of getting prostate cancer will also affect the probability of progressing to advanced disease once prostate cancer has developed.

Using a two-step procedure described in supplemental method, we looked for prognostic correlation between clinical properties and SNP-expression associations. In one case, association 40 (*FOXD1* and rs9623117 C), the penetrance of the association appears to extend to an association with survival. This association is consistent with the properties of *FOXD1*. *FOXD1* is a member of Forkhead family of transcription factors involved in the Wnt pathway [51], which regulates epithelial-mesenchymal transition (EMT) in cancer. EMT is an important mechanism by which prostate cancer gains aggressive properties in cell migration, vascular invasion and early metastases. Moreover, Koga *et al*. characterized *FOXD1* as a mediator and indicator of the cell reprogramming process, the prevention of *FOXD1* expression resulted in a reduced number of iPSCs (induced pluripotent stem cells) [52]. Thus the properties of *FOXD1* suggest one testable mechanism that explains the association of the *FOXD1*-rs9623117 C with survival observed here.

Multiple trans-associations between SNPs and gene expression in prostate cancer have not been observed before. Our results provide new ways for understanding the basis of risk for eight SNPs previously associated with occurrence of prostate cancer, namely involving altered expression among identified genetically unlinked genes. In future studies, it will be desirable to expand the number of samples in our study to more reliably test parameters associated with progression. It will also be desirable to obtain both expression and SNP data from archived normal stroma samples that have many years of follow-up data on the subsequent occurrence of prostate cancer. Such samples may reveal the role of SNPs in regulating expression in stroma not only after cancer has occurred, as we have studied here, but also during the onset of prostate cancer.

## MATERIALS AND METHODS

### Prostate cancer patient samples

Tissues for a total of 55 patients treated by radical prostatectomy (RP) for prostate cancer were analyzed. Informed consent was obtained in all cases following a protocol approved by the UCI Office Research Administration Institutional Review Board (IRB) as part of the NCI “SPECS” consortium at UCI for Strategic Partners for the Evaluation of Cancer Signatures for Prostate Cancer. All tissues were collected at surgery and escorted to pathology for expedited review, dissection and snap freezing in liquid nitrogen.

Demographic and clinical parameters such as Pre-operative Prostate Specific Antigen (Pre-PSA), surgical margin status, post-prostatectomy Gleason sum, age, T stage, are presented in Supplementary Table S1. Biochemical relapse was defined as post-operative PSA >0.2 ng/ml following a post-operative PSA nadir of undetectable for patients with surgical negative margins.

### DNA and RNA preparation

The frozen tissue of each subject was manually microdissected while mounted in a cryostat into multiple sections for RNA/DNA preparation. Tissue for RNA preparation was monitored by frozen section preparation and examination of sections with hematoxylin and eosin stains to ensure the location of tumor enriched tissue. Frozen tissue was directly dissolved in TRIzol® Reagent. RNA was prepared from stroma adjacent to tumor and used for expression analysis as previously described [31, 53]. DNA was prepared from prostate tissue remote from tumor for hybridization to Illumina Human 1M-Duov3 B arrays exactly as recommended by Illumina Inc.

### Expression, genotyping, and eQTL analysis

80 RNA samples from 55 patients, providing two or three biological replicates for some patients, were hybridized to Affymetrix GeneChip U133plus2 for gene expression analysis. The raw intensity data was background corrected, normalized, and summarized to be gene expression data through RMA (Robust Multi-array Average) algorithm [54]. The resulting Microarray data have been deposited in the publicly accessible Gene Expression Omnibus (GEO) database with accession number GSE17951. Purified DNA samples of 55 patients were applied to Illumina Human1M-Duov3_B SNP array for genotyping. In eQTL analysis, we selected 49 tissue samples containing more than 50% stroma. The tissue composition for each patient sample that has been used for array assay (tumor epithelial cells, stroma cells, epithelial cells of BPH) was determined by four pathologists [23, 55].

In order to identity associations between SNPs and transcript levels, we modified the BAYES [34] method to perform eQTL analysis. The BAYES method is a model-based iterative method that analyzes all expressed transcripts and SNPs jointly. The method combines multiple linear regression (MLR) with unsupervised clustering analysis in order to identify associations between transcripts and SNPs. The MLR method is the backbone of the model which describes the relationship between transcripts and SNPs with the assumption that expression level of each transcript is the sum of contributions of all possible relevant SNPs (Eqn. 1).

$$y_{ij}=\beta_{j}+\sum_{k=1}^{Q}Z_{ik}\gamma_{jk}+\varepsilon_{ij}$$ (1)

Where *y_ij_* is the expression level of transcript *j* for the *i*th patient, *β_j_* is the intercept for transcript *j*, *Z_ik_* is the genotype of SNP *k* for the *i*th patient, *γ_jk_* is the effect of the SNP *k* on transcript *j* (i.e., a measure of SNP-expression association), *ε_ij_* is the residual error. In each iteration, the unsupervised clustering analysis is applied to cluster the transcripts into SNP-associated clusters and non-SNP-associated clusters for each SNP. A positive association is observed between a transcript and a SNP if the transcript is clustered into the SNP-associated group after *n* iterations with more than 80% of iterations supporting the association. The advantage of BAYES method is that a transcript may be simultaneously associated with multiple SNPs and *vice versa*. In order to reduce the computing time, we modified the BAYES method by applying the Stochastic Expectation-Maximization (SEM) algorithm instead of the Monte Carlo Markov Chain (MCMC) algorithm in the model. The simulation study showed that the two algorithms achieve the same result upon eQTL analysis.

## SUPPLEMENTARY MATERIALS


