# Supplementary material for: The identification of trans-associations between prostate cancer GWAS SNPs and RNA expression differences in tumor-adjacent stroma
Source: Oncotarget. 2015 Feb 9;6(3):1865–73. doi: 10.18632/oncotarget.2763 (PMC4359337; doi:10.18632/oncotarget.2763)
Supplement: Supplementary file 1 [file oncotarget-06-1865-s001.pdf]

SUPPLEMENTARY MATERIALS

METHOD: TWO-STEP PROCEDURE

The two-step procedure was performed for 47 transcript-SNP associations separately. The first step is to cluster patients into three risk groups. The second step is to apply ordinal logistic regression analysis to examine the associations between three risk groups and clinical properties in unadjusted univariate and multivariate models. The linkage and the clinical property are said to be associated if the *p*-value from the two-step procedure is significant. Below we describe the first step in details.

Step 1: For each transcript-SNP linkage, we define group 1 as patients with risk allele of the SNP, and define group 2 as patients with non-risk allele of the SNP. The boxplots of transcript expression were generated for group 1 and 2, respectively (Supplementary Figure S1). A comparison between the boxplots was made to determinate the up or down-regulation of the transcript. The up-regulated transcript represents the median of transcript expression in group 1 is higher than the median of transcript expression in group 2. For up-regulated transcripts, patients with expression values in the top half of the transcript expression in group 1 are defined as high risk group. Patients with expression values in the bottom half of the transcript expression in group 2 are defined as low risk group. The remaining patients are defined as intermediate risk group. For down-regulated transcripts, patients with expression values in the bottom half of the transcript expression in group 1 are defined as high risk group. Patients with expression values in the top half of the transcript expression in group 2 are defined as low risk group. The remaining patients are defined as intermediate risk group.

REFERENCES

1. Eeles RA, Kote-Jarai Z, Al Olama AA, Giles GG, Guy M, Severi G, Muir K, Hopper JL, Henderson BE, Haiman CA, Identification of seven new prostate cancer susceptibility loci through a genome-wide association study. Nat Genet. 2009; 41:1116–1121.
2. Thomas G, Jacobs KB, Yeager M, Kraft P, Wacholder S, Orr N, Yu K, Chatterjee N, Welch R, Hutchinson A, Multiple loci identified in a genome-wide association study of prostate cancer. Nat Genet. 2008; 40:310–315.
3. Gudmundsson J, Sulem P, Gudbjartsson DF, Blondal T, Gylfason A, Agnarsson BA, Benediktsdottir KR, Magnusdottir DN, Orlygsdottir G, Jakobsdottir M, Genome-wide association and replication studies identify four variants associated with prostate cancer susceptibility. Nat Genet. 2009; 41:1122–1126.
4. Duggan D, Zheng SL, Knowlton M, Benitez D, Dimitrov L, Wiklund F, Robbins C, Isaacs SD, Cheng Y, Li G, Two genome-wide association studies of aggressive prostate cancer implicate putative prostate tumor suppressor gene DAB2IP. J Natl Cancer Inst. 2007; 99:1836–1844.
5. Sun J, Zheng SL, Wiklund F, Isaacs SD, Purcell LD, Gao Z, Hsu F-C, Kim S-T, Liu W, Zhu Y, Evidence for two independent prostate cancer risk-associated loci in the HNF1B gene at 17q12. Nat Genet. 2008; 40:1153–1155.
6. Amundadottir LT, Sulem P, Gudmundsson J, Helgason A, Baker A, Agnarsson BA, Sigurdsson A, Benediktsdottir KR, Cazier JB, Sainz J, A common variant associated with prostate cancer in European and African populations. Nat Genet. 2006; 38:652–658.
7. Yeager M, Orr N, Hayes RB, Jacobs KB, Kraft P, Wacholder S, Minichiello MJ, Fearnhead P, Yu K, Chatterjee N, Genome-wide association study of prostate cancer identifies a second risk locus at 8q24. Nat Genet. 2007; 39:645–649
8. Eeles RA, Kote-Jarai Z, Giles GG, Al Olama AA, Guy M, Jugurnauth SK, Mulholland S, Leongamornlert DA, Edwards SM, Morrison J, Multiple newly identified loci associated with prostate cancer susceptibility. Nat Genet. 2008; 40:316–321
9. Xu J, Zheng SL, Isaacs SD, Wiley KE, Wiklund F, Sun J, Kader AK, Li G, Purcell LD, Kim ST, Inherited genetic variant predisposes to aggressive but not indolent prostate cancer. Proc Natl Acad Sci USA. 2010; 107:2136–2140.
10. Gudmundsson J, Sulem P, Steinthorsdottir V, Bergthorsson JT, Thorleifsson G, Manolescu A, Rafnar T, Gudbjartsson D, Agnarsson BA, Baker A, Two variants on chromosome 17 confer prostate cancer risk, and the one in TCF2 protects against type 2 diabetes. Nat Genet. 2007; 39:977–983.
11. Gudmundsson J, Sulem P, Rafnar T, Bergthorsson JT, Manolescu A, Gudbjartsson D, Agnarsson BA, Sigurdsson A, Benediktsdottir KR, Blondal T, Common sequence variants on 2p15 and Xp11. 22 confer susceptibility to prostate cancer. Nat Genet. 2008; 40:281–283.
12. Sun J, Zheng SL, Wiklund F, Isaacs SD, Li G, Wiley KE, Kim S-T, Zhu Y, Zhang Z, Hsu F-C, Sequence variants at 22q13 are associated with prostate cancer risk. Cancer Res. 2009; 69:10–15.

SUPPLEMENTARY FIGURES AND TABLES

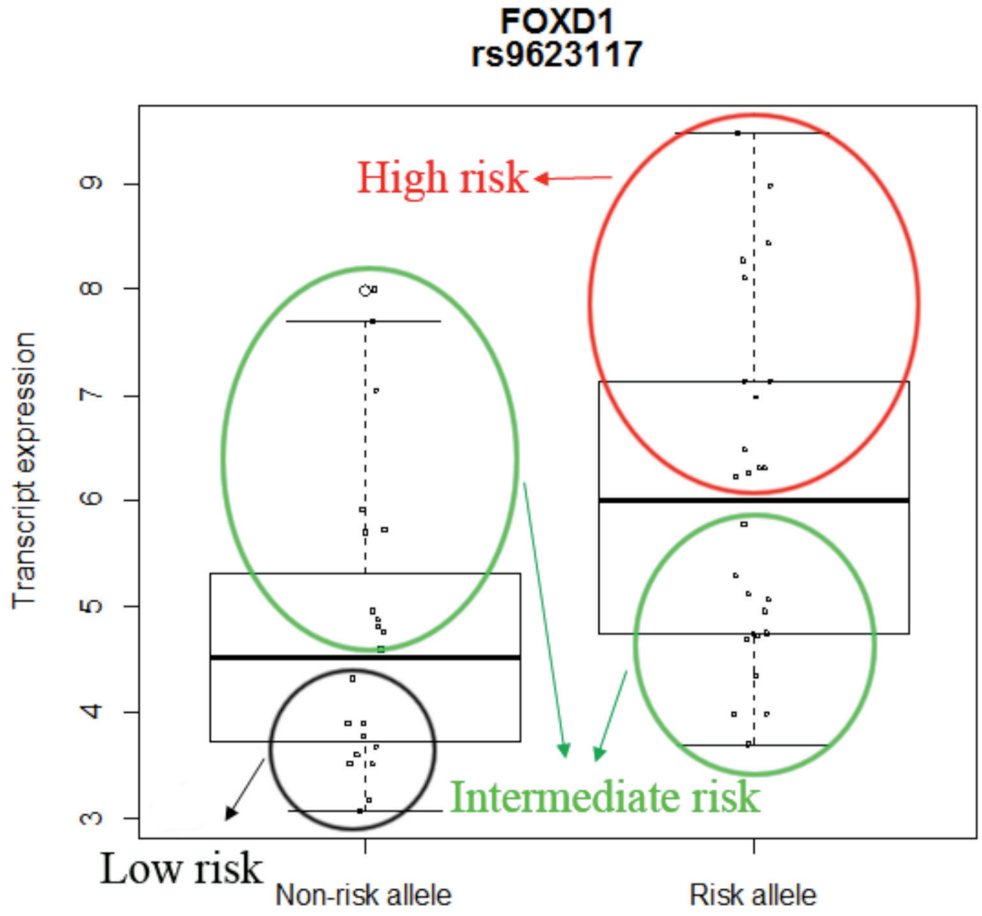

Supplementary Figure S1: Three risk groups clustered by *FOXD1*-rs9623117. The clustering procedure is described in supplementary method two-step procedure in details.

Supplementary Table S1: Clinical and pathologic tumor characteristics of 49 patients.

	Data Set
No. of patients	49
Age (years), mean (SD)	60.7 (5.9)
Preoperative PSA (ng/ml)	6.7
Average Follow up (Months)	24.0
Biochemical Recurrence	
Relapse	20 (40.8%)
Non-relapse	27 (55.1%)
Unknown	2 (4.1%)
Gleason Sum	
3–6	17 (34.7%)
7	26 (53.1%)
8–10	6 (12.2%)
Pathological Stage	
T1	1 (2.0%)
T2	35 (71.4%)
T3	11 (22.5%)
T4	0 (0.0%)
Unknown	2 (4.1%)
Surgical Margins	
Negative	30 (61.2%)
Positive	18 (36.7%)
Unknown	1 (2.0%)

Supplementary Table S2: Summary of 35 SNPs information analyzed in this study.

SNP ID	Location	Risk/non-risk Allele	Reference	Sample Size (case *vs*. control)
rs10086908	8q24	C/T	[1]	5499 vs 5833
rs10486567	7p15	G/A	[2]	4760 vs 5133
			[1]	3650 vs 3940
			[3]	1725 vs 35392
rs10896449	11q13	G/A	[2]	4760 vs 5133
			[1]	5501 vs 5830
rs10896450	11q13	C/T	[1]	5500 vs 5828
rs10934853	3q21	A/C	[4]	1235 vs 1599
			[3]	13774 vs 47614
rs10993994	10q11	T/C	[2]	4760 vs 5133
			[1]	5502 vs 5832
			[3]	1727 vs 35397
rs11228565	11q13	A/G	[3]	7185 vs 7215
rs11649743	17q12	G/A	[5]	9446 vs 7214
			[2]	4760 vs 5133
			[3]	1747 vs 35405
rs12500426	4q22	A/C	[1]	21731 vs 20650
rs12621278	2q31	G/A	[1]	21721 vs 20655
rs1447295	8q24	A/C	[6]	3430 vs 2375
			[7]	4296 vs 4299
			[2]	4760 vs 5133
			[1]	5504 vs 5834
			[3]	1821 vs 35470
rs1465618	2p21	A/G	[1]	21688 vs 20599
rs1512268	8p21	A/G	[1]	21732 vs 20654
rs1571801	9q33	A/C	[4]	1032 vs 571
rs17021918	4q22	T/C	[1]	21506 vs 20567
rs1859962	17q24	G/T	[1]	5449 vs 5734
			[3]	1746 vs 35124
rs2660753	3p12	T/C	[8]	5097 vs 5228
			[3]	1725 vs 35362
rs2735839	19q33	G/A	[1]	5501 vs 5826
			[3]	1726 vs 35376
rs2928679	8p21	C/T	[1]	21724 vs 20649
rs401681	5p15	C/T	[3]	1962 vs 35400
rs4054823	17p12	T/C	[9]	4784 vs 12093

(*Continued*)

SNP ID	Location	Risk/non-risk Allele	Reference	Sample Size (case *vs*. control)
rs4430796	17q12	A/G	[10]	3490 vs 14345
			[8]	1613 vs 1798
			[2]	4760 vs 5133
			[5]	9334 vs 7064
rs4962416	10q26	C/T	[2]	4760 vs 5133
rs5759167	22q13	T/G	[1]	21732 vs 20654
rs5945572	Xp11	A/G	[11]	10054 vs 28879
			[1]	5499 vs 5831
rs5945619	Xp11	C/T	[1]	5499 vs 5831
rs620861	8q24	C/T	[1]	5277 vs 5746
rs6465657	7q21	C/T	[1]	5499 vs 5828
			[3]	1724 vs 35358
rs6983267	8q24	G/T	[7]	4296 vs 4299
			[2]	4760 vs 5133
			[1]	5487 vs 5813
			[3]	1724 vs 35367
rs7127900	11p15	A/G	[1]	21726 vs 20642
rs721048	2p15	A/G	[11]	10093 vs 28654
rs7931342	11q13	G/T	[1]	5502 vs 5829
			[3]	1951 vs 35394
rs8102476	19q13	C/T	[2]	4760 vs 5133
			[3]	13173 vs 47198
rs9364554	6q25	T/C	[1]	5500 vs 5829
			[3]	1725 vs 35399
rs9623117	22q13	C/T	[12]	4755 vs 7200

Supplementary Table S3: The summary results between SNP-expression associations and clinical properties in ordinal logistic regression analysis.

	Proportional Odds Ratio	95% CI(low)	95% CI(high)	*P* Value
**Age**				
Association 36				
Univariate model	1.11	1.01	1.23	0.044
Multivariate model	1.13	1.01	1.29	0.040
Association 38				
Univariate model	1.12	1.01	1.24	0.040
Multivariate model	1.21	1.06	1.40	0.006
Association 39				
Univariate model	1.15	1.03	1.29	0.015
Multivariate model	1.27	1.11	1.52	0.002
**Biochemical Relapse**				
Association 6				
Univariate model	3.05	0.98	10.16	0.060
Multivariate model	4.22	1.13	17.72	0.039
Association 40				
Univariate model	4.73	1.45	17.65	0.014
Multivariate model	4.55	1.19	19.68	0.032
